# Visual salience of the stop signal affects the neuronal dynamics of controlled inhibition

**DOI:** 10.1038/s41598-018-32669-8

**Published:** 2018-09-24

**Authors:** Pierpaolo Pani, Franco Giarrocco, Margherita Giamundo, Roberto Montanari, Emiliano Brunamonti, Stefano Ferraina

**Affiliations:** 1 Department of Physiology and Pharmacology, Sapienza University, Rome, Italy; 2 Behavioral Neuroscience PhD Program, Sapienza University, Rome, Italy

## Abstract

The voluntary control of movement is often tested by using the countermanding, or stop-signal task that sporadically requires the suppression of a movement in response to an incoming stop-signal. Neurophysiological recordings in monkeys engaged in the countermanding task have shown that dorsal premotor cortex (PMd) is implicated in movement control. An open question is whether and how the perceptual demands inherent the stop-signal affects inhibitory performance and their underlying neuronal correlates. To this aim we recorded multi-unit activity (MUA) from the PMd of two male monkeys performing a countermanding task in which the salience of the stop-signals was modulated. Consistently to what has been observed in humans, we found that less salient stimuli worsened the inhibitory performance. At the neuronal level, these behavioral results were subtended by the following modulations: when the stop-signal was not noticeable compared to the salient condition the preparatory neuronal activity in PMd started to be affected later and with a less sharp dynamic. This neuronal pattern is probably the consequence of a less efficient inhibitory command useful to interrupt the neural dynamic that supports movement generation in PMd.

## Introduction

Many daily decisions that we make are conditioned by the efficiency with which our brain processes sensory stimuli. For example, at a traffic light, our ability to stop after a red signal could be strongly affected by the presence of distractors (e.g., a new sound in the environment) or by a decrease in the signal-to-noise ratio of the signal against the background (e.g., a high-intensity ambient light).

Many studies have used perceptual tasks to examine decision processes. Typically, in a controlled experimental setting, subjects are presented with various amounts and qualities of visual information, and their performance, evaluated as their choice or response time, reflects the amount and consistency of the information that is accumulated. In general, with larger amounts of available information (or lower levels of uncertainty), the response becomes faster and more accurate. When incorporating neurophysiological approaches, studies suggest that a decision is taken when a signal of evidence reaches a threshold level in support of the action that will be made^1–4^.

The frontal and parietal cortical areas of the primate brain, when studied in decision tasks, contain neurons, the activity of which shows evidence of accumulation dynamics^3,5–10^, combined eventually with an urgency-to-respond process^11^. In some of these areas (e.g., the lateral intraparietal area [LIP] and the dorsal premotor cortex [PMd]), evidence of sensorimotor transformations emerges—for instance, sensorial information is integrated into a movement preparation activity that is later transformed into an action. Changes in neuronal activity typically correlate with the difficulty to perceive a stimulus, choices, and response times. For example, the easier it is to detect a stimulus or to distinguish between alternatives, the faster the evidence accumulates and the shorter the response time. The uncertain relationship between a target and an action can also be expressed by tasks in which multiple actions are programmed simultaneously, only one of which is selected, based on a delayed instructional cue^12^.

In many perceptual tasks, decisions are communicated with an action, an overt behavior—typically a hand or eye movement. A similar outcome is unavailable when the perceptual task triggers a decision for the cancellation or execution of an action, as in the response to the red traffic signal above. The absence of direct behavioral evidence is an important reason why we know much less about the processes that support the decision to suppress a behavior. This lack of knowledge remains, despite the ability to suppress being central in many fields of neuroscience, undergoing alterations in various neuropsychiatric and neurological diseases^13,14^.

To examine this issue directly, recent behavioral studies used modified versions of the countermanding task, which has been used extensively to evaluate the suppression abilities at the neuronal and behavioral levels^15,16^. In this task, the subject is required to respond to a Go stimulus in most of the trials (no-signal trials) but must halt the response when an unpredictable Stop signal follows after varying delays (stop-signal delay, SSD) in a subset of trials (stop-signal trials). In each stop-signal trial, one can withhold (signal-inhibit trials) or generate the response (signal-respond trials). This task allows one to evaluate the so-called reactive inhibition, which is described by the probability to respond to the Stop signal as a function of the SSD lengths and by the duration of the stop process (stop-signal reaction time, or SSRT). The latter measure is an estimate of the time that it takes to stop the response after presentation of the Stop signal^15^. The SSRT can be broadly considered to be the response time of the inhibitory decision process. As such, it is supposed to be influenced by various task demands, including the presence of distractors, the modality/intensity of the Stop signal, and changes in the focus of attention^17–26^. However, no direct neurophysiological studies have been performed to describe how perceptual demands influence inhibitory decisions in motor-related areas.

In the frontal lobe of primates, the PMd is important in visuomotor transformations for decision-making. The neuronal activity of this area represents various properties of the impending movement, including its direction, distance, trajectory, timing, and speed^27–39^, and it signals the direction of potential and final reach choices^12^ and the selection of specific rules^40^. Further, it reflects conflicts that are related to the dynamic competition between contemporary alternative movement choices^41,42^ and expresses a decision process that is related to the selection of actions that are based on sensory cues^43^, dynamic sensory signals^42^, and changes in one’s mind^44^. In animals that are trained in the countermanding task, the PMd shows neuronal activity that modulates according to movement inhibition. These neuronal modulations have been described on various scales, and the evidence demonstrates that changes in single-unit activities (SUAs), local field potentials (LFPs), and multiunit activities (MUAs) predict whether a movement will be correctly suppressed after an unexpected Stop signal^45–48^.

Using a perceptual countermanding task that we recently validated in humans^19^, we found that less salient Stop stimuli deteriorate inhibitory performance and correspond to longer SSRTs in non-human primate subjects.

Further, these behavioral data were subtended by neuronal modulations in the PMd, examined on the MUA scale, that began later and showed a less steep dynamic for stimuli that were difficult to be processed during successful suppressed movements.

## Results

### Behavioural performance and neuronal data selection

**Figure 1:**
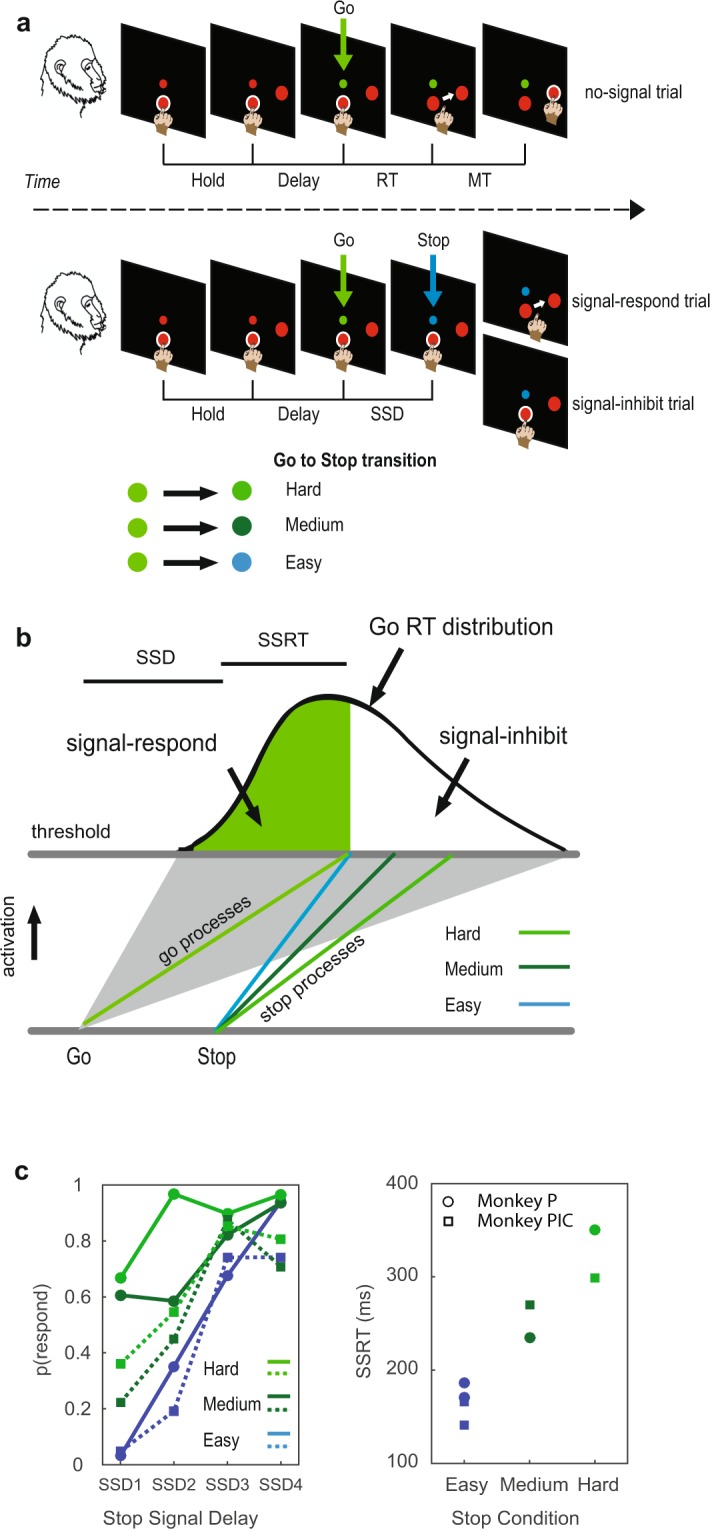
Countermanding task (multi-stop-signal version) description and behavioural results. (**a**) Every trial started with the simultaneous appearance of the central target (large red circle) and of the cue signal (small red circle). Monkeys touched the central target (Hold time, variable duration). After, the peripheral target appeared and the Delay period started. Then the change in colour of the cue signal was used as Go, instructing for a reaching movement towards the peripheral target. In no-signal trials the monkeys were rewarded upon the touch of the peripheral target. In stop-signal trials the monkeys had to refrain from moving to get the reward (signal-inhibit trials); otherwise, if a movement was made, the reward was not delivered (signal-respond trials). One out of three different stop-signals (a further change in colour of the cue) could unpredictably and equally probable appears (Go to Stop transition: easy, medium, hard). The white halo around either the central or the peripheral target was used as feedback of touch for the monkey. (**b**) Schematic of the race model to illustrate the two processes racing toward the threshold in stop-signal trials. The go process is shown as mean (green line) and the possible range (corresponding to the full distribution of RT). The stop processes are indicated separately for the three conditions assuming a slope effect. (**c**) Effects of the stop-signal salience modulation on inhibitory performance: Left column: Inhibition functions obtained for the two monkeys (Monkey P: circles; Monkey PIC: squares) in the fixed SSDs session; Right column: SSRTs values for each monkey and condition (see Behavioral Results and Table 1 for details). RT, reaction time. SSRT, stop signal reaction time. SSD, stop signal delay.

In the stop-signal trials of the perceptual countermanding task we adopted (Fig. 1a) the Stop signal corresponded to a change in colour and brightness, detectable with different levels of difficulties (*easy*, *medium*, or *hard*), of the Go signal after a variable SSD.

Using the countermanding task, two indices of inhibitory control could be derived from the behavior: the first is the average probability of responding on signal trials [p(respond), i.e., the percentage of signal-respond trials in the session], and the second is the SSRT, computed using the integration method^15^ (see Materials and Methods for further details). In the example considered here for the *easy* stop condition (Fig. 1b, blue line for the stop process), the curve depicts the distribution of RTs on no-signal trials, representing the finishing times of the go process (green line for the mean value and grey area for the full range). In the specific case illustrated, the mean of the go process bisects the go RT distribution, meaning that the go arrival time is simultaneous to the finishing time of the stop process of the *easy* stop condition. Given that the response could not be stopped on the 50th percent of all stop trials (signal-respond; green region), SSRT is calculated by subtracting the mean SSD from the 50th percentile of Go RT.

The effect of the stop process with reduced efficiency is shown (Fig. 1b), assuming a change in the slope for simplicity. Given their delayed arrival to the threshold, they should correspond to a reduced amount of signal-inhibit trials [increased p(respond)] and to an elongation of the SSRT duration (*medium* and *hard* conditions). In fact, for both animals (Monkeys P and PIC) the average p(respond) and the SSRT values increase with the increased amount of difficulty on the Go-to-Stop transition (Fig. 1c). However, the delayed arrival to the threshold can be caused by other factors, like the delayed onset of the stop process, either in isolation or in combination with a change in the slope.

For the neuronal data analysis, we selected two behavioral sessions for each animal— one session for the fixed-SSD procedure and one for the tracking procedure (see Materials and Methods) — both respecting the behavioral assumptions of the race model (see Materials and Methods) and consisting of more than 1000 total trials per session (in order to have a sufficient proportion of signal-inhibit and signal-respond trials for each condition).

**Figure 2:**
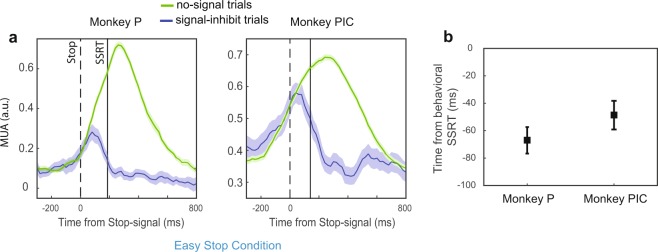
Neuronal modulation in signal-inhibit trials. (**a**) For the easy condition, activity comparison between signal-inhibit trials and latency-matched no-signal trials (single movement direction; population data). Modulation is evident before the end of the SSRT. Data are relative to a single recording session (Monkey P, n = 28 electrodes; monkey PIC n = 6 electrodes). (**b**) Latencies of the divergences between signal-inhibit and no-signal trials (mean ± SE) relative to the finish time of the stop process (SSRT) (monkey P, n = 58; monkey PIC n = 12).

For the neuronal analysis, we decided to describe the changes in the MUA modulation, obtained from the unfiltered signal (see Materials and Methods), recorded from each electrode in PMd (see Supplementary Fig. S1) and processed as previously described^47^. Briefly, MUA was estimated as the change in power of the Fourier high-frequency components from the unfiltered signal. We initially selected data from electrodes with reaching-related (see Materials and Methods) MUA modulation, in no-signal trials and in at least one of the two directions of movement. From this dataset, we further selected those channels related to the countermanding task—i.e., we selected MUA modulations in the PMd predicting whether or not a movement will be made (see Fig. 2a for the modulation at the population level and Supplementary Fig. S2 for single-channel examples) by evaluating their time of divergence between no-signal and signal-inhibit trials (Fig. 2b; see Material and Methods). For this last selection, we employed only signal-inhibit trials in the *easy* conditions: indeed, across monkeys and sessions, this condition provides reliable estimates of SSRTs (see Behavioral Results); furthermore, previous neurophysiological studies have employed salient stop-signals that were easy to detect to establish the role of neuronal activity in movement control (as such roughly corresponding to the *easy* condition)^16,45,46,49,50^.

In conclusion, we focused on the modulation of 70 electrodes (58 for Monkey P, 30 and 28 in two separate sessions [selected from 65 and 57 channels with movement-related modulation, respectively], and 12 for Monkey Pic, 6 and 6 in two separate sessions [selected from 15 and 13 channels with movement-related modulation, respectively]), sampling neuronal activity directly involved in the action control (by expressing a reaching-related preparatory activity and modulation in the SSRT). In the following paragraph, we will describe how this modulation was affected by the different Stop signals used.

### The salience of the Stop signal affects PMd neuronal dynamics in signal-inhibit trials

To investigate the effect of Stop signal salience on neuronal activity, we compared *easy*, *medium*, and *hard* signal-inhibit trials aligned to the stop-signal presentation.

**Figure 3:**
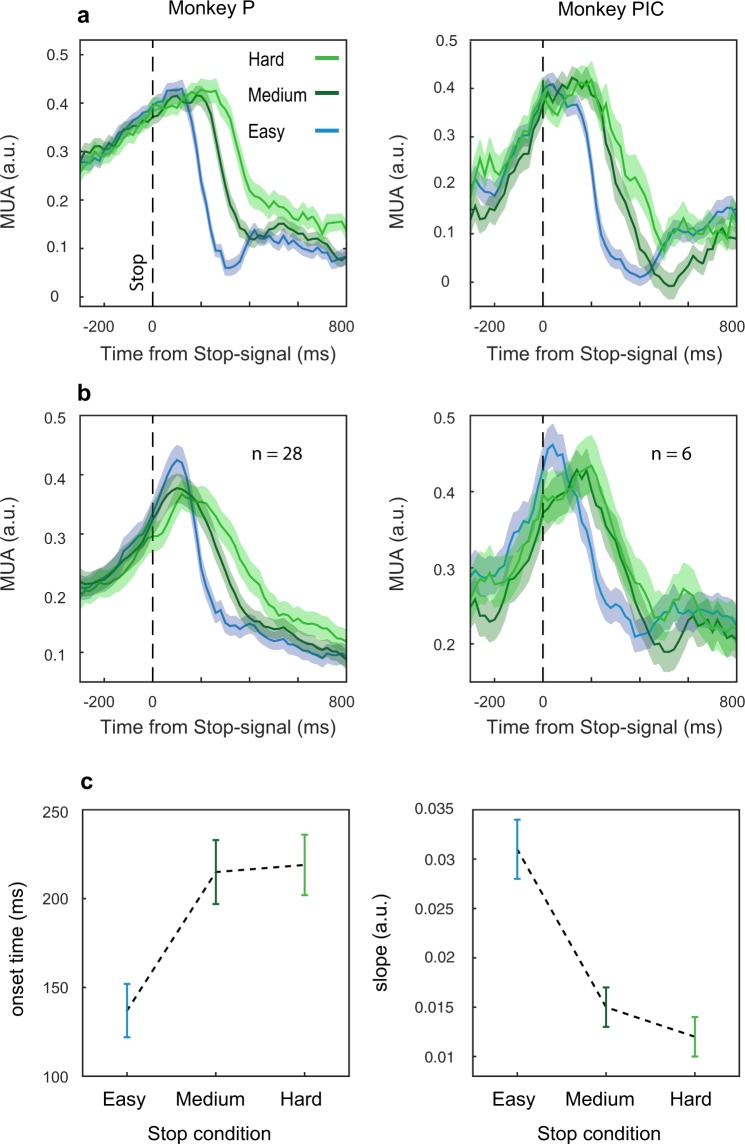
Effect of salience conditions on MUA modulation in signal-inhibit trials. Neuronal activity is aligned to the Stop signal and it is represented for a single channel (**a**) and at the population level for a single session (**b**) separately for each monkey (monkey P, for the population n = 28; monkey PIC, for the population n = 6). (**c**) Average ( ± SE) onset times and slopes of the neuronal modulations across all sessions and monkeys.

For both monkeys, we found clear differences between the salience conditions both for single recording channels as well at the population level (see Fig. 3a,b). When the Stop-signal was *easy* to detect, the suppression of neuronal activity started at about 130 ms after the Stop signal presentation; differently, it was delayed for the *medium* and *hard* conditions (up to about 220 ms). Furthermore, the neuronal dynamics appear to be different: in the *easy* conditions, compared to the others, a steeper suppression occurred (Fig. 3c).

Statistical testing supported the phenomenological pattern of neuronal activities (Fig. 3): two separate ANOVAs—one for the latency of the onset of suppression relative to the Stop-signal (onset times) and the other for the slopes, with factors monkeys and stop-signal conditions—were done. For the slope analysis, we found that the slopes changed depending on the salience (F(2, 116) = 32.4, p = 0.00000): the *easy* condition showed a steeper slope (mean = 0.031; 95 CI = 0.024, 0.037) compared to the *medium* and the *hard* conditions (*medium*: mean = 0.015; 95 CI = 0.011, 0.019; *hard*: mean = 0.012; 95 CI = 0.008, 0.016; Newman- Keuls post hoc test: MSE = 0.00012, df = 116, *easy* vs *medium* p = 0.0001; *easy* vs *hard* p = 0.0001); however, *medium* and *hard* were not different between each other (p = 0.45).

The analysis of the onset times showed that salience conditions affected the inhibition process (F(2, 114) = 12.231, p = 0.00002; Newman-Keuls post hoc, MSE = 0.00680, df = 114): specifically, in *easy* trials, MUAs were suppressed earlier (mean = 136, 95 CI = 105, 167) than in *medium* and *hard* conditions (*medium*: mean = 214, 95 CI = 178, 251; p = 0001; *hard*: mean = 218, 95 CI = 183 254, p = 0.0001, respectively); however, no differences were detected between *medium* and *hard* conditions (p = 0.4).

Thus, in general, we found that the higher the salience of the Stop-signal, the earlier the modulation of neuronal activity started and the stronger (steeper) the modulation observed. This is in line with the behavioral results. We considered as the behavioral index of the inhibitory performance the p(respond) for the same sessions employed for the neuronal data analysis. We found a significant effect of conditions on the behavior (F(2,18) = 11.531, p = 0.0006). Further, the inhibitory performance reflected the stop-signal salience well *(easy*: mean = 0.48; 95 CI = 0.25, 0.70; *medium*: mean = 0.64; 95 CI = 0.49, 0.80; *hard*: mean = 0.74; 95 CI = 0.6, 0.88. Newman-Keuls post hoc test: MSE = 0.01523, df = 18, *easy* vs *medium* p = 0.008; *easy* vs *hard* p = 0.0006): indeed, in the *easy* conditions, we observed fewer errors compared to the *medium* and to the *hard;* however, we did not find a significant difference between *hard* and *medium* (p = 0.1), as observed in the neuronal data.

We also performed correlational (and regression) analysis between the SSRT values estimated in the behavioral analysis and the onset times and the slopes of the neuronal activity. We decided to perform a multiple linear regression analysis (stepwise method) using onset times and slopes as predictors and SSRTs as observed responses. We found that the predictors together contributed to explain 89% of the variance (F(2,5) = 29.65; p = 0.002; standardized beta coefficients: slopes = −0.648, (p = 0.004); onset times = 0.561 (p = 0.007)). Both neuronal indexes were correlated with SSRTs (estimated Pearson correlation coefficient: onset times = 0.728 (p = 0.02); slopes −0.79 (p = 0.009)). The slopes alone could account for about 57% of the variance, with the onset times adding about 32% in the model. Thus, these analyses show that there is a strong relationship between the indexes of neuronal activity and the behavioral performance.

### Lack of interference between the stop process and the go process in signal-respond trials

**Figure 4:**
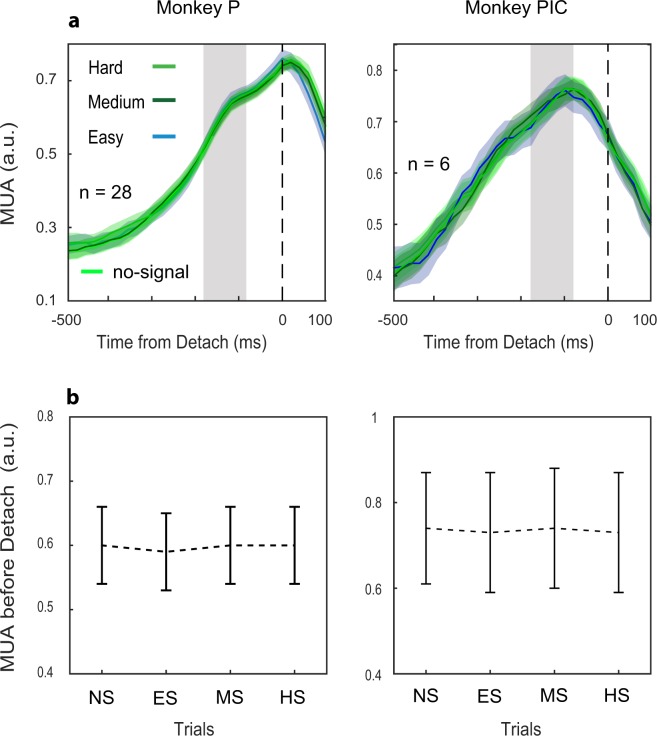
Comparison between no-signal and signal-respond trials. (**a**) Population average for all conditions (latency-matched no-signal trials; hard/medium/easy Stop signal for the signal-respond trials) and sessions (Monkey P, tracking SSD; Monkey PIC, fixed SSD). The grey areas show the ‘before detach’ epoch of analysis. (**b**) Average activities (mean ± SE) in the ‘before detach’ epochs for data in a.

In the behavioral analysis, we found that the Race model’s independence assumption (signal-respond trials RTs must be shorter than the no-signal trials; see Materials and Methods) was confirmed for the *easy* conditions but not always for the *medium* and *hard*. One possible explanation is that in the *medium* and *hard* conditions, the stop process interacted with the go process lengthening RT in signal-respond trials. An alternative explanation is that the higher length of the stop process in *medium* and *hard* conditions gives the possibility to have longer RTs in signal-stop trials, thus making it more difficult to find differences between no-signal and signal-respond trials. We already found evidence at the neuronal level that the inhibitory process is affected in the *medium* and *hard* condition. However, this effect could be combined with an interference effect, or, in other terms, both altered neuronal dynamics and interference can participate in producing longer signal-respond RTs. To evaluate whether some interference was active in signal-respond trials, we compared the activity of signal-respond trials, separately for each condition, to latency-matched no-signal trials (see Materials and Methods). If an inhibitory process had been active in signal-respond trials, the corresponding neuronal activity should have been lower than the selected no-signal control trials, where no inhibitory process was active. To this aim, we considered as latency-matched the no-signal trials with RTs shorter than the average SSD of all signal-respond trials plus the SSRT estimated for each session in the *easy* condition. We then calculated the average MUA from −180 to −80-ms to movement onset (Fig. 4; Detach). We also considered the same interval for all the signal-respond trials in each condition. We found a three-way interaction between the factors considered (monkeys, sessions, and type of trial: F(3, 198) = 6.5, p = 0.00034); thus, we ran Dunnet post-hoc tests between each pair of no-signal and signal-respond trials. We report the data (mean (SE)) separately for each monkey and session: Monkey P, fixed SSDs: no-signal 0.46(0.06); signal-respond: *easy* 0.46(0.06), *medium* 0.46(0.06), *hard* 0.51(0.06), (all p’s > 0.63); Monkey P, tracking SSDs: no-signal 0.6(0.06); signal-respond: *easy* 0.59(0.06), *medium* 0.6(0.06), *hard* 0.6(0.06), (all p’s > 0.8); Monkey PIC, Fixed SSDs: no-signal 0.74(0.13); signal respond: *easy* 0.73(0.14), *medium* 0.74(0.14), *hard* 0.73(0.14), (all p’s > 0.62); Monkey PIC, tracking SSDs: no-signal 0.37(0.14); signal respond: *easy* 0.38(0.14), *medium* 0.39(0.14), *hard* 0.37(0.14), (all p’s > 0.95); (MSE = 0.111, df = 66.297). These data show that the failure of the independence assumption for signal-respond trials was not supported by suppressed activation of neuronal activity. To further strengthen our observation, we performed the same comparison by considering all no-signal trials. Qualitatively, identical results were obtained (three-way interaction analyzed by means of Dunnet post hoc tests: F(3, 198) = 3.9, p = 0.009, all p’s of post-hoc comparisons >0.8).

Thus, we can exclude that at least at the level of the PMd, interference between stop and go processes can explain the failure of the independence assumption and the lengthening of signal-respond trials (see also Fig. 4).

## Discussion

The aim of this study was to examine the neuronal activity in the PMd with regard to perceptual decisions during the inhibitory process. We used a countermanding task in which the salience of the Stop signal was modulated: we found that higher salience was associated with a greater ability to stop. This behavioral performance was predicted well by the modulation in the preparatory neuronal activity. Following a well-detectable Stop signal, compared with Stop signals that were less salient, we observed modulation of neuronal activation that began earlier and showed a steeper slope, suggesting a “faster” stop-related decision process.

This is the first behavioral and neurophysiological study to explicitly combine the perceptual modulation of the stop command in the context of the countermanding task.

The temporal patterns that we observed are reminiscent of studies on the neuronal mechanisms that underlie decisions that involve perceptual processing and movement selection. For example, studies that use Random-Dot Motion^51^ (RDM) require saccades to be made toward the target that corresponds to the direction in which the highest fraction of dots is coherently moving. Increasing the fraction of dots that move coherently and, thus, the strength of the stimulus simplifies the task. The accuracy increases and the RT decreases for stimuli that are easy to decode. Neuronal activity in parietal areas (e.g., the LIP) reflects, with ramp of activities having different slopes, the decision process concerning where and when to move, based on the fraction of coherent motion that is detected: the stronger the stimulus, the steeper the accumulation of evidence and the shorter the RT^2^.

There are similarities between our task and the pattern above. We did not have the equivalent of a random-dot Stop signal, but the modulation of the visual salience of the Stop signal clearly affected the inhibitory performance. If we consider the decrease in activity that we observed as a reflection of an active process against the generation of a movement, we can see that its temporal evolution parallels the signal that reflects the typical accumulation of evidence.

However, the latency of the activity build-up is not affected in RDM discrimination tasks. Other studies have shown that onset times are good predictor of RTs. For example, one study^52^ examined how movement-related activity in the Frontal Eye Fields (FEF) of macaques is affected by the decoding of perceptual information in a visual search task. In a version of this task, monkeys had to select a target among distracters and make a saccade toward it. The authors used 2 conditions: efficient search and inefficient search. In the first condition, detection of the target was easy. In the second, by introducing distracters that were closer to the target in colour or space, the detection was more difficult, as evidenced by the resulting RTs and errors. They found that under the inefficient condition, in most recorded neurons, the movement-related activity began later compared with the efficient condition. This study suggests that the onset of movement activity is a neuronal parameter that predicts behavioral performance under various perceptual conditions. The function of onset time in predicting differences in RTs has been confirmed in other studies^4^.

We found that both the slopes and onset times of neuronal activity are modulated during movement suppression, thus presenting a combination of the phenomena that have been observed in the RDM task and other perceptual decision-making studies.

As a limitation of the present study we have to stress that we are observing the effect of the Stop signal strength indirectly, in the modulation of the preparatory activity. The effects observed on the preparatory activity can be subtended by different, not mutually exclusive, neuronal dynamics. For example, as illustrated (Fig. 1b), the change in preparatory activity as function of the Stop signal salience can be supported by a change in the slope of the stop process. A weaker rise of the stop process can account for a delayed and a less steeper modulation, as observed in the medium and hard conditions. Alternatively, a similar effect can be related to an elongation of the encoding phase of the Stop signal, corresponding to a late onset of the rise. Finally a combination of the two dynamics is possible. Examining the dynamics of the stop command to distinguish between alternative hypotheses would require recordings in a similar task from the prefrontal cortex or basal ganglia region, which are likely the source of the inhibitory process^13^.

In recent years, several groups have examined the function of the PMd in motor decision using tasks in which the sensorial instruction required a form of discrimination (eg, establishing which of two colours was more predominant^41,43^) or changed continuously^11^. In these studies, neuronal activity reflected the process that was related to the formation of the decision with or without the accumulation of evidence^42^. For example, in a study that measured cortical laminar differences in processing on a decision-making task, a subpopulation of neurons in the PMd superficial layers showed a build-up of neuronal activity that was steeper for easy and faster responses, thus reflecting the decision process^43^.

Thus, these studies support the hypothesis that the pattern that we observed reflects a decision-to-stop process that is modulated by visual salience.

Previous neurophysiological studies on movement inhibition in primates, through the countermanding task, recorded neuronal activity from the FEF and SC and used an easy-to-detect Stop-signal^16,49^. The same type of Stop-signal was obtained in studies that determined the neuronal correlates of the inhibition of arm movements^45,50^. Concurrently, behavioral studies have shown that sensory features of the Stop signal and the presence of distractors affect inhibitory performance^19–23,53,54^. Further, simulation studies have examined the function of the detection process in permitting the inhibition of saccades^55^ and how response inhibition results from blocking the input to the go process, thus impeding its growth to generate the movement. However, no direct neurophysiological experiments have been performed. Our work is seminal, in that it demonstrates the neuronal consequences of the modulation of perceptual processing during movement inhibition, providing new insights into the refinement of neuronally inspired models of movement control^55–58^.

Another important contribution of our results is that it confirms the function of the PMd as a site of movement suppression. We have shown that MUAs convey a signal that is sufficient to predict whether a movement will be inhibited, as observed for single neurons^45^ and as suggested by movement planning dynamics in delayed reaching tasks^47^.

Regarding the high temporal and spatial definition of neurophysiology, PMd is the only cortical area that shows such a strong relationship with arm movement suppression. With the PMd, the SMA experiences slow field potential modulations in temporal relation with movement inhibition in the countermanding task. However, these field potentials can be related to widespread phenomena that likely reflect an incoming signal from other regions (such as the prefrontal cortex and basal ganglia), as observed in other contexts^59–64^. Whether this signal will be used to stop the movement or regulate other aspects of the behavior that is related to the task could be determined by considering the MUAs or the firing rates of single cells^65^. We hypothesize that the PMd provides the output to other cortical or subcortical structures, as suggested in other neurophysiological and neuroanatomical studies^39,66–73^.

Our data support the function of the PMd in movement inhibition, because the neuronal modulation under the various conditions covaried with behavioral performance. Although our data are correlational in nature, support to a role of PMd comes also from TMS studies^74–78^ as well from lesions in both monkeys and humans^79,80^.

The function of the PMd in humans has also been confirmed by electrocorticography^47^ and supported in part by fMRI studies^81,82^. It is like that the nature of the signal and the wide temporal definition make it difficult to extract a signal that is related to the neuronal dynamics of this region.

Behaviorally, performance in monkeys approximates that in humans^19^, suggesting that the same neuronal processes are involved. This possibility is particularly notable when considering the application of basic neurophysiological knowledge to help clinical investigations. We must still detail the neuronal processes that support the decision to suppress a behavior: this lack of knowledge exists, despite the central nature of the ability to suppress in many fields of neuroscience—it appears to be damaged in various neuropsychiatric and neurological diseases^13,83,84^. For example, a detriment in inhibition performance is often observed in persons with attention deficit hyperactivity disorder (ADHD) in the stop task^54,85–87^. However, the specific components that are affected are unknown^88,89^. Considering our results, several mechanisms could participate in reducing the ability to control the movement, such as a diminished triggering of the stop response, a delayed onset of stop implementation due to a longer encoding phase, and a slow rate of implementation of the stop process.

Neurophysiologically inspired models could help predict specific behavioral performance, based on the malfunction of various components, thus increasing our understanding of the neurocognitive basis of neuropsychiatric and neurological disorders.

## Materials and Methods

### Subjects

Two male rhesus macaque monkeys (Macaca Mulatta, Mon P and PIC) weighing 9 and 13 kg were employed for this study. Monkeys were pair-housed with cage enrichment. They were fed daily with standard primate chow, supplemented with nuts and fresh fruits if necessary. The monkeys received their daily water supply during the experiments. All experimental procedures, animal care, housing, and surgical procedures conformed to European (Directive 2010/63/UE) and Italian (D.L. 26/2014) laws on the use of nonhuman primates in scientific research and were approved by the Italian Ministry of Health.

### Animal preparation

A single 96-channel Utah array (BlackRock Microsystem, USA) was implanted in the PMd (using anatomical landmarks, arcuate sulcus - AS - and pre-central dimple - pCD) of each monkey. The site of the implant was contralateral to the arm used during the experiment. All the surgeries were performed under sterile conditions and veterinary supervision. Antibiotics and analgesics were administered postoperatively. Anesthesia was induced with ketamine (Imalgene, 10 mg kg^−1^ i.m.) and medetomidine hydrochloride (Domitor, 0.04 mg kg^−1^ i.m. or s.c.) and maintained by inhalation isoflurane (0.5–4%) in oxygen (5 l/min). Antibiotics were administered prophylactically during surgery and postoperatively for at least 1 week. Postoperative analgesics were given at least twice per day. Recordings started well after recovery from surgery (after a minimum of 10 weeks). A head-holding device was implanted in monkeys PIC before training, while in monkey P the headholder was implanted simultaneously with the array (see below).

### Apparatus and tasks

Experiments were performed in a darkened acoustic insulated room. Monkeys were seated in front of a black isoluminant background (<0.1 cd/m2) of a 17-inch touchscreen monitor (LCD, 800 × 600 resolution). A non-commercial software package, CORTEX (ftp://helix.nih.gov/lsn/matoff/), was used to control stimuli presentation and behavioral responses.

Figure 1a shows the schema of the general task employed, which is a modified version of the reaching countermanding task^21,45,47,65,90,91^, characterized by the presentation of two types of trials, randomly intermixed: no-signal trials and stop-signal trials (see Table 1).

**Table 1:** Detailed behavioral results.

Monkeys Session	n trials	% Stop	no-signal RT Mean(SD)	s-r RT Easy Mean(SD); p	s-r RT Medium Mean(SD); p	s-r RT Hard Mean(SD); p	SSRT Easy	SSRT Medium	SSRT Hard
P(fixed)	1228	33	637.9(126.1)	589(114); p = 0.001	642(136); p = 0.9	638(112); p = 0.9	171(84)		
P(tracking)	1399	36	565(104)	526(82); p < 0.001	548(95); p = 0.2	562(122); p = 0.4	186	235	350
Pic(fixed)	1131	27	627(177)	518(154); p < 0.0001	569(120); p = 0.0042	569(158); p = 0.0042	166(68)	270(122)	299(114)
Pic(tracking)	1517	30	387(126)	357(133); p < 0.3	397(158); p = 0.5	412(186); p = 0.5	141		

For different monkeys and sessions, the following data are reported: overall number of trials (n trials); percentage of Stop signals presented (% Stop); mean and SD of reaction times (RTs) for no-signal, signal-respond (s-r) easy, medium, and hard trials together with the p value from the comparison between no-signal and the target signal-respond trial groups. Stop signal reaction times (SSRTs) are reported only for comparisons for which signal-respond RTs were not nominally higher than no-signal RTs.

Each trial started with the appearance of a central target (CT) (red circle, diameter 1.9 cm) and a Cue signal (red circle, diameter 0.7 cm) slightly above (3 cm) the CT at the center of the screen. Monkeys had to touch the CT and maintain their finger on it.

After a variable delay (500–800 ms, 50-ms step), a peripheral target (PT) (red circle, diameter 1.9 cm) appeared randomly in one out of two possible locations (i.e., 7 cm at the right or left of the screen vertical midline for Monkey P; only at the right for monkey PIC in one session).

In no-signal trials, after a foreperiod delay [fixed duration (120 ms) in monkey P; 800–1200 ms (50-ms steps) in monkey PIC], a Go stimulus, consisting of a green circle, appeared, replacing the Cue (no-signal condition; stimulus circle 0.7 cm RGB: [0 250 0]; 85 cd/m2). The Go stimulus instructed the subjects to reach the peripheral target as fast as possible and to hold the new position for a variable time (600–800 ms), until the end of the trial. Reaction times (RTs) were defined as the time between the Go stimulus presentation and the hand movement onset towards the PT. An upper temporal limit (upper reaction time) was set at 1200 ms. Delaying the response behind this limit would correspond in aborted trials. This value was set during the training to instruct the monkey to respond fast, avoiding waiting for the Stop signal. During the recording session, all no-signal RTs were below this limit.

In stop-signal trials, the sequence of events was exactly the same until the Stop signal (circle, 0.7 cm) replaced the Go stimulus after a variable delay (stop-signal delay or SSD). In these trials, the simple detaching of the hand after the Go stimulus presentation corresponded to a wrong response.

Conversely, a hand kept on the CT until the end of the trial (800–1000 ms, 50-ms step) corresponded to a correct response (signal-inhibit trial; Fig. 1, lower right panel).

For both no-signal and stop-signal trials, movements performed before the Go stimulus aborted the trial, and trials were considered as not engaged and excluded from further analysis. For correct no-signal trials and signal-inhibit trials, monkeys experienced a brief sound accompanied by the delivery of the juice reward. In signal-respond trials, neither sound nor reward was delivered, and the screen became blank.

The inter-trial interval was set at 800 ms. Three different types of equiprobable and intermingled Stop-signals, distinguishable in colour and brightness, could follow the Go stimulus, depending on the stop-signal condition. These Stop signals were classified as *easy*, *medium*, and *hard* (stimulus circle 0.7 cm SS_easy [0 0 188], 7 cd/m2; SS_medium [0 160 0], 44 cd/m2; SS_hard [0 210 0], 66 cd/m2), being progressively closer to the Go stimulus in terms of appearance (see Fig. 1a).

Two different procedures were adopted to establish the duration of the SSDs: tracking and fixed. By using the tracking procedure, we controlled the duration of the SSDs on the basis of the performance in the last stop-signal trial: if the monkey succeeded in withholding the response, the SSD increased by one step (150 ms, for both monkeys) in the subsequent stop-signal trial. Conversely, if the subject failed, the SSD decreased by one step. Each stop-signal condition had its own independent staircase procedure. By using the fixed SSD procedure, we employed 4 different SSDs (from 170–620, with a 150-ms step, for both monkeys). The same SSDs were employed for each of the Stop signals.

### Behavioral Analysis

The performance in the countermanding task is accounted by the race model—in stop trials, two stochastic processes race toward a threshold: the go process triggered by the onset of the Go signal, and the stop process triggered by the onset of the Stop signal. The result of this race, either movement generation in signal-respond trials or movement suppression in signal-inhibit trials, will depend on which of these processes will reach its own threshold first. In signal-inhibit trials, the stop process wins over the go process, and vice versa. By making the SSDs unpredictable and variable, the output of the race is affected: the longer the SSD, the higher the probability to facilitate the go process. To make the employment of the race model fruitful to study response inhibition, a central assumption must be satisfied: the go process in the stop-signal trials must be the same as in the no-signal trials (independence assumption^15,92^). To broadly validate this assumption, signal-respond trial RTs must be shorter than the no-signal trials^93^. All the sessions included in this study respected this prediction for at least one stop-signal condition—typically, the easy condition (see Results). Once the independence assumption has been validated, Stop-signal Reaction Time (SSRT) can be estimated using two main variables: the reaction time (RT) distributions of no-signal trials and signal-respond trials, and the probability to respond (respond) by error to the Stop signal. Following recent recommendations^93^, we first confirmed that the independent assumption was respected. Because our behavioral analysis was performed at the single-session level, we adopted comparison criteria between average values, because in these conditions, the employment of a statistical test can be too strict a criterion^93^. However, in the easy conditions, for 3 out of 4 sessions, the difference was also statistically confirmed (rank-sum test, see Appendix of Results). Then, we proceeded in calculating the SSRT by using the integration method^92^. The method assumes that the finishing time of the stop process corresponds to the *n*th no-signal RT, where *n* results from the multiplication of the ordered no-signal RT distribution by the overall probability of responding, p(respond), when using the tracking procedure. The SSRT can then be calculated by subtracting the mean SSD from the *n*th no-signal RT. When the fixed procedure was employed, we applied the same method at each SSD (provided the *p*(respond) was above 0 and below 1). As in previous similar approaches^16,45,49^ we then averaged SSRTs to obtain a single value.

### Neuronal recordings and analysis

Neuronal activities were recorded from 96-channel Utah arrays (BlackRock Microsystem, USA) by using specific software (Tucker Davies Technologies, unfiltered raw signal, sampling rate 24.4 kHz). Array data in this paper come from two recording sessions for both monkey P (2-month interval between sessions) and monkey PIC (6-month interval). We used recordings sufficiently separated in time in order to reduce oversampling from the same population of neurons. The multiunit activity (MUA) that we extracted is a good approximation of the average firing rate as described in detail in^47^. In this study, we smoothed the signal obtained by using a moving average sliding window (±40-ms sliding window, 20-ms step). We selected channels displaying a reaching-related MUA modulation for at least one movement direction—i.e., a significant difference between the activity preceding the movement onset (from −250 to −50 ms before detachment) and the baseline (from 0 to 100 ms following the touch of the CT). This type of modulation is an index that neuronal activity recorded from that channel is potentially involved in movement preparation and control. For the main analysis reported here, we focused on channels in which neuronal activity was modulated before the end of SSRT, as done in other works in primates on the same topic^16,45,46,49,50^.

To compare neuronal activities between signal-inhibit and no-signal trials, we considered a subsample of the last group of trials: the so-called latency-matched trials. These are trials in which RTs were longer than the specific SSD (or average SSD) plus the corresponding behavioral SSRT.

When no-signals were compared to signal-responds, the latency matching was performed by considering the no-signal trials with RTs longer than 100 ms and shorter than SSD + SSRT. Latency-matched trials are trials that have a similar level of movement preparation of the target trials for the comparison (either signal-inhibit or signal-respond).

To select neuronal responses involved in movement suppression, we established whether MUA modulation could predict movement inhibition by estimating when the divergence between signal-inhibit and latency-matched trials (potentially suppressed as in Fig. 1b) started. To gain more power, we aligned the trials to the Stop signal presentation, considering the average SSD for the latency-matched trials. We calculated a differential MUA function by subtracting the average MUA in signal-inhibit trials in the easy condition from the latency-matched no-signal trials. We defined the time at which the differential MUA exceeded the mean difference in the 200 ms preceding the Stop signal presentation by 2 SD and stayed above this limit for at least 120 ms as time of the divergence.

From the values obtained, we subtracted the corresponding SSRT and obtained a negative value, indicating the time lag between neuronal and behavioral estimates of inhibition. Negative values indicate that the neuronal modulation occurs before the behavioral estimate.

To evaluate the latency of the MUA modulation following the Stop signal, we identified the time from the stop presentation at which MUA began to show a decreasing trend that went on for at least 100 consecutive ms. This time was defined as the onset time. Starting for the onset time, we considered the following 200 ms, and we ran a robust regression function (Matlab, robustfit) to extract the slope of the MUA suppression pattern.

This analysis was repeated for each selected channel and stop condition across all the recording sessions for both monkeys. The datasets generated and/or analyzed for the current study are available from the corresponding author on reasonable request.

## Electronic supplementary material

Supplementary Information
