# Supplementary material for: Visual salience of the stop signal affects the neuronal dynamics of controlled inhibition
Source: Sci Rep. 2018 Sep 24;8:14265. doi: 10.1038/s41598-018-32669-8 (PMC6155270; doi:10.1038/s41598-018-32669-8)
Supplement: Supplementary file 1 — Supplementary Information [file 41598_2018_32669_MOESM1_ESM.pdf]

Supplementary Information

Visual salience of the stop-signal affects neuronal dynamics of controlled inhibition

Pierpaolo Pani[1]*[§], Franco Giarrocco[12]*, Margherita Giamundo [1], Roberto Montanari [1], Emiliano Brunamonti [1], Stefano Ferraina [1]

*=equally contributed

[§]=corresponding author

[1]Department of Physiology and Pharmacology, Sapienza University

[2] Behavioral Neuroscience PhD program Sapienza University

Correspondence

Pierpaolo Pani PhD

Department of Physiology and Pharmacology

P.le Aldo Moro 5 - 00185 Rome, Italy

+39064991772

Email: pierpaolo.pani@uniroma1.it

Supplementary Figure S1

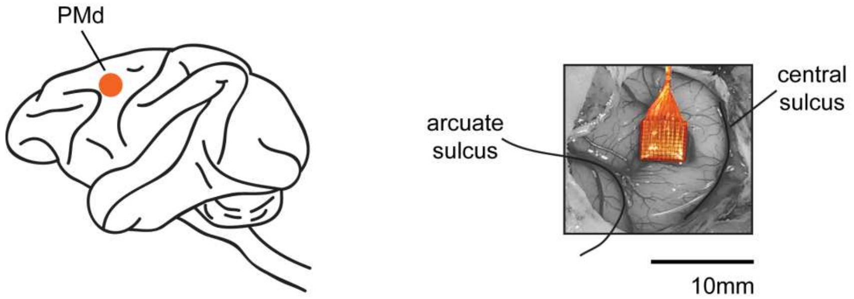

Recording site. Schematic of the location of the arrays in the cortex. (left) Location was similar between animals as controlled by visual inspections of the anatomical landmarks (arcuate sulcus, central sulcus, precentral dimple) during surgery. (right) Detailed picture of the array in monkey P.

Supplementary Figure S2 (related to Figure 2 in the main text)

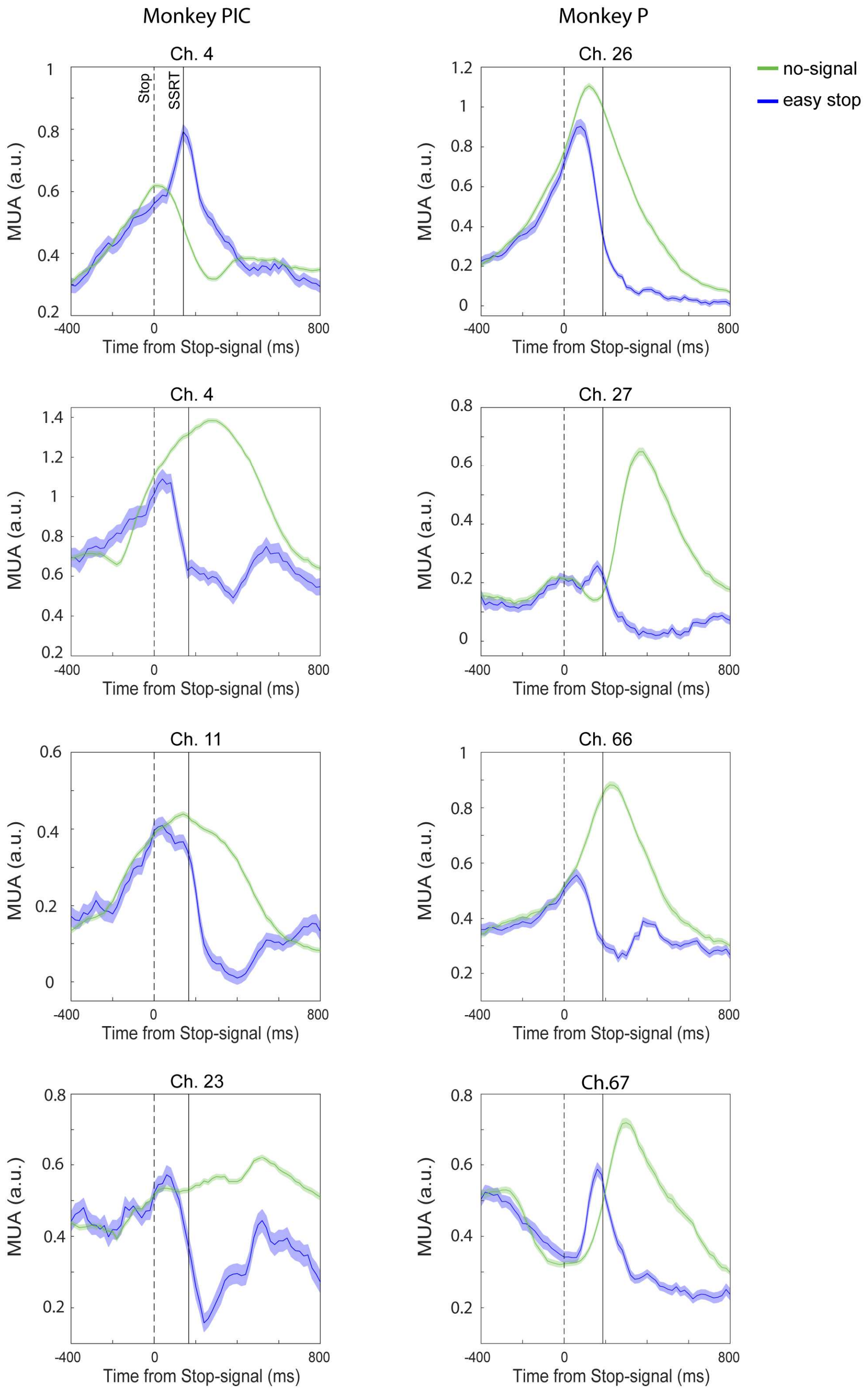

Comparisons between signal-inhibit trials (*easy* stop condition) and latency-matched no-signal trials for single channels (Ch) and for both monkeys. Channel 4 is presented twice to show the different modulation in the two sessions used as an example of the selection criteria

Supplementary Figure S3

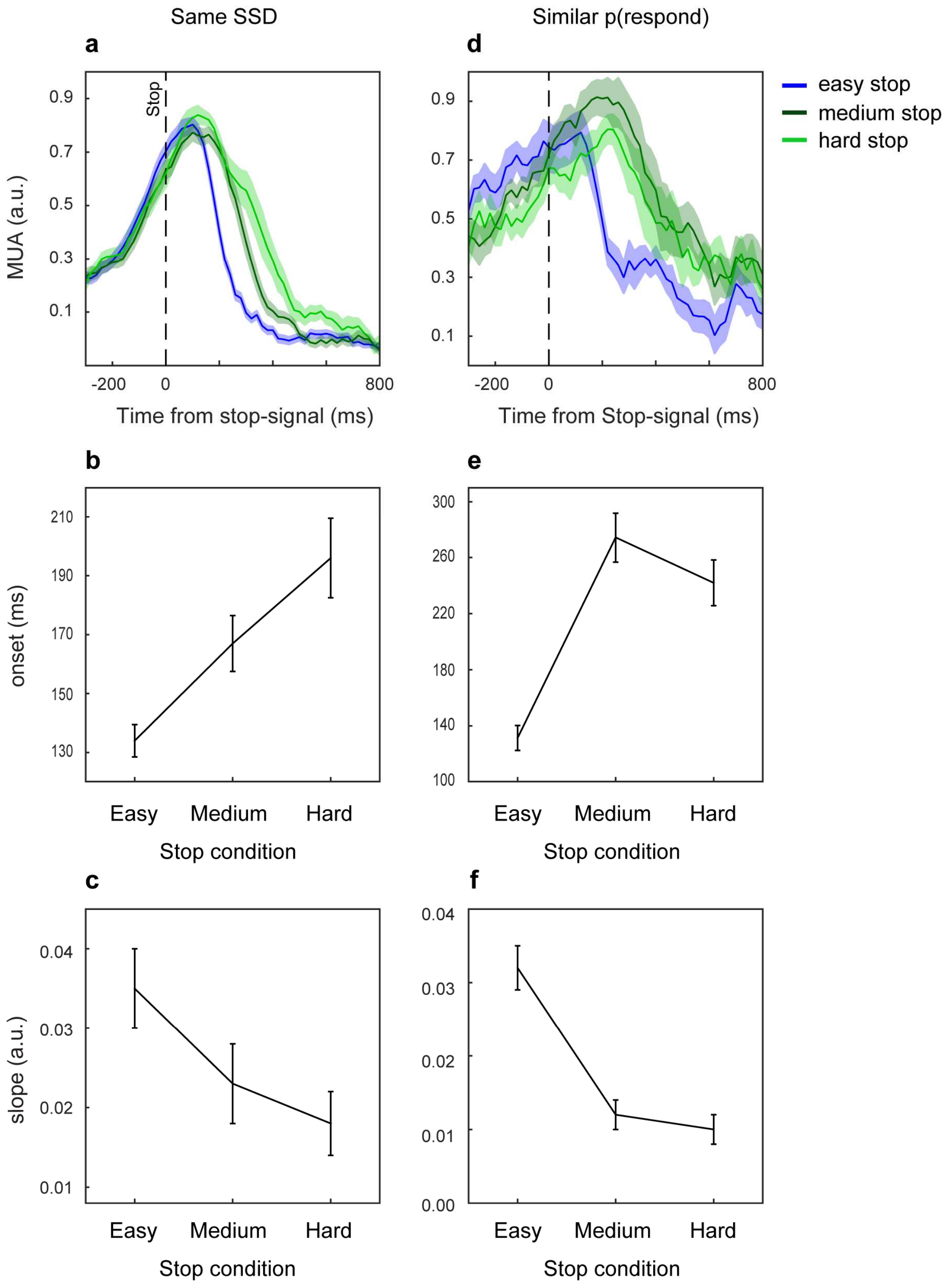

Neural dynamics after Stop-signal presentation for specific group of trials. (**a**) For an example channel (Mon. P; staircase SSD), average (±SE) neuronal modulation for signal-inhibit trials selected for the same SSD value; Population average (±SE; n= 28) of onset time (**b**) and slope (**c**) modulation for signal-inhibit trials selected for the same SSD value; (**d**) For an example channel (Mon. P; fixed SSD), average (±SE) neuronal modulation for signal-inhibit trials selected for similar p(respond) values; Population average (±SE; n= 30) of onset time (**e**) and slope (**f**) modulation for signal-inhibit trials selected for similar p(respond) values.

Analysis of stop performance for specific group of trials

Because the analysis described in the main text were conducted at the population level, one could ask whether the patterns we described are the consequence of the averaging among trials corresponding to different SSDs or different probability of responding. To control for these potential factors, we extracted two different group of trials: signal-inhibit trials for which the length of the SSD was the same and a group of trials for which the p(Respond) were similar. Aligning to the same SSD, we confirmed that the neural patterns showed different slopes and onset times depending on the condition (Slope comparison: n=27; F(2, 52)=14.8 p=.000001; mean(SE): *easy*=0.035(.005); *medium*=0.023(.005); *hard* =0.018(0.004); Onset times comparison: n=27; F(2, 52)=25.2, p=.00000; mean (SE): *easy*=134(5.5); medium=167(9.5); hard=196(13.5), see Supplemental Figure 3)

If the different conditions are characterized by a different neural dynamics, the diversity should be confirmed also by the analysis for the conditions with a similar probability of response (easy= 0.68; medium =0.59; hard= 0.67; Monkey P, fixed SSD session); Statistical test confirmed the finding (Slope comparison (n=21; F(2, 40)=25.02, p=.00000; mean(SE) *easy* (0.032(.003); (0.012(0.002); *hard* (0.01 (0.002)). The same dynamic was observed for the latency of the onset of the modulations (F(2, 40)=38,893, p=,00000; *easy* 131.4(8.9); *medium* 274.3(17.6); *hard* 241.9(16.3), see Supplemental Figure 3)

This control analyses corroborated the evidence that visual salience influenced the neural patterns related to movement inhibition by changing both the onset time of the modulation (when the stop process acts on preparatory neural activity) and the slope at a small sample level, as already found at the population level.

Supplementary Figure S4

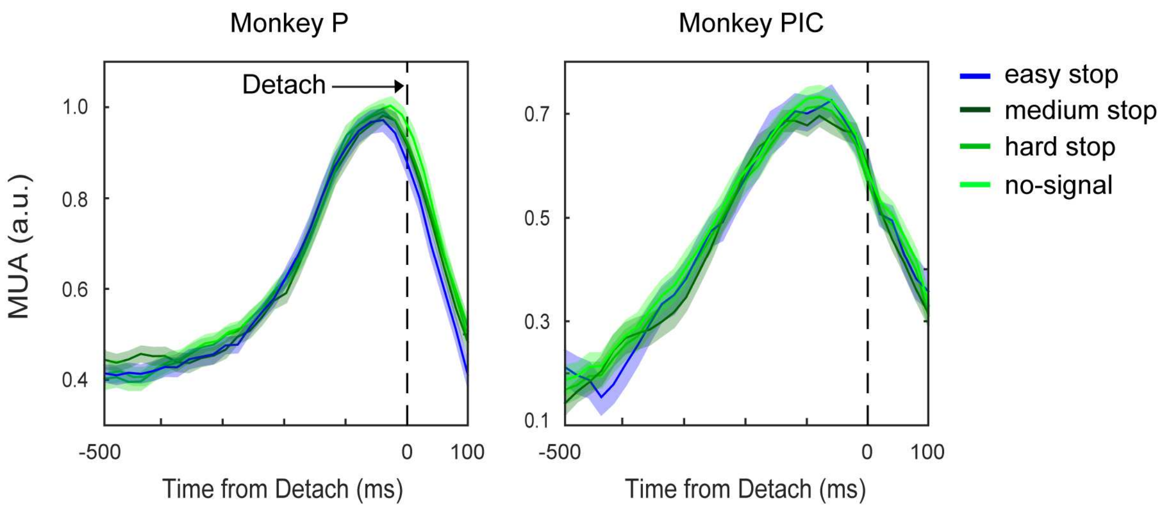

Comparison between average activity (±SE) of no-signal and signal-respond trials for single example channels and for both monkeys.
